# Recent Progress on Triboelectric Nanogenerators for Vibration Energy Harvesting and Vibration Sensing

**DOI:** 10.3390/nano12172960

**Published:** 2022-08-26

**Authors:** Ahmed Haroun, Mohamed Tarek, Mohamed Mosleh, Farouk Ismail

**Affiliations:** Department of Mechanical Design and Production Engineering, Cairo University, Giza 12613, Egypt

**Keywords:** triboelectric nanogenerator, vibration energy harvesting, hybrid generators, triboelectric displacement sensors, triboelectric acceleration sensors

## Abstract

The triboelectric nanogenerator (TENG) is a recent technology that reforms kinetic energy generation and motion sensing. A TENG comes with variety of structures and mechanisms that make it suitable for wide range of applications and working conditions. Since mechanical vibrations are abundant source of energy in the surrounding environment, the development of a TENG for vibration energy harvesting and vibration measurements has attracted a huge attention and great research interest through the past two decades. Due to the high output voltage and high-power density of a TENG, it can be used as a sustainable power supply for small electronics, smart devices, and wireless sensors. In addition, it can work as a vibration sensor with high sensitivity. This article reviews the recent progress in the development of a TENG for vibration energy harvesting and vibration measurements. Systems of only a TENG or a hybrid TENG with other transduction technologies, such as piezoelectric and electromagnetic, can be utilized for vibrations scavenging. Vibration measurement can be done by measuring either vibration displacement or vibration acceleration. Each can provide full information about the vibration amplitude and frequency. Some TENG vibration-sensing architectures may also be used for energy harvesting due to their large output power. Numerous applications can rely on TENG vibration sensors such as machine condition monitoring, structure health monitoring, and the Internet of things (IoT).

## 1. Introduction

**Figure 1:**
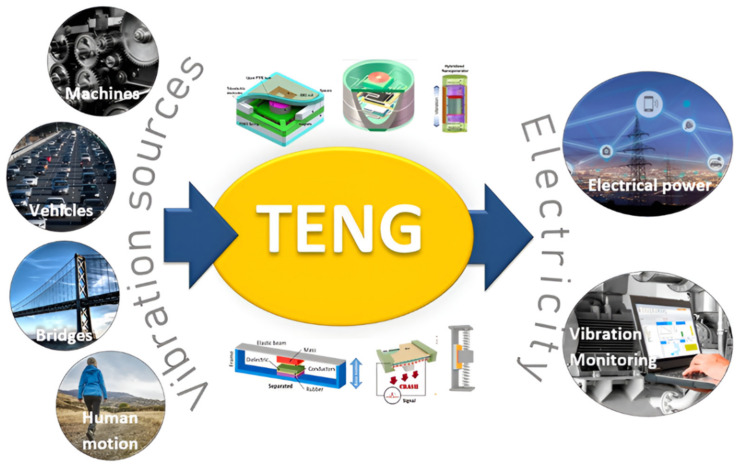
TENG can convert mechanical vibrations into electricity, which can be used for electrical powering or vibration sensing applications.

Energy harvesting, which is the process of capturing and converting dissipated energy from the surrounding into electricity [1], has become an attractive field of research. It is considered as an effective self-powering technique for wireless sensors. Several forms of wasted energy exist in the surrounding environment such as wind energy, solar energy, and vibrational energy. Mechanical vibrations are an abundant source of energy [2,3] that appear in everyday life (Figure 1) in vehicles [4,5], structures such as buildings and bridges [6], machines [7], etc. Vibration energy harvesting can be an effective powering strategy for wireless sensors that are placed in deep and dark locations, where there is no exposure to air or light [8,9]. Three main technologies of energy transduction have been used for years to convert mechanical energy into electricity, which are piezoelectric [10,11,12,13,14,15,16,17], electromagnetic [18,19,20,21,22,23,24], and electrostatic [25,26,27,28,29,30]. A newly developed electrostatic technology that can convert mechanical energy into electricity has been introduced by Wang’s group in 2012, which is the triboelectric nanogenerator (TENG) [31,32,33]. TENG technology is based on combination between contact electrification and electrostatic induction [34]. It has been introduced as a promising technology to scavenge mechanical energy from many available sources [35] in the ambient environment such as vibrations [36,37,38,39], human motion [39,40], waves [41,42], and wind [43,44]. The triboelectric nanogenerator shows some advantages when compared with other technologies, as it exhibits a large output voltage [45], high efficiency [46], low cost [47], a simple fabrication method [48], great broadband behavior, excellent robustness, reliability [49], and eco-friendliness, as well as efficient low-frequency vibration energy scavenging [32,33,34,35,47]. According to those features of a TENG as well as the low power consumption of modern wireless sensors, a TENG can be a sustainable power source (Figure 1) for many wireless-sensing applications [50,51].

Coupling a TENG with other generator types, such as piezoelectric (PEG) and electromagnetic (EMG) generators, to form a hybrid is also introduced [52]. A hybrid generator can take the advantages of multiple generator types and enhance the output performance at different input motion conditions. For example, an EMG—TENG can provide higher output voltage, higher output current, and wider bandwidth than each generator separately [53].

The same concept of converting mechanical vibrations into electricity is also applied for vibration sensing (Figure 1). A self-powered vibration sensor can detect vibration signals without the need for a battery as a power unit. Vibration measurement is essential for many applications. Machine-condition monitoring is one of the important applications that allows the detection/prediction of machine damages, abnormal stoppings, and disasters. The accurate monitoring of a machine’s condition and process is very essential to enhance the feedback system and optimize the operation. Some transduction technologies have been developed for vibration sensing, such as magnetoresistive [54,55], piezoresistive [55], piezoelectric [56], etc. Each can be fitted to specific applications [57].

A TENG can transfer vibrations or other kinds of motion such as triggering, rotational motion, linear displacement, or physical motion into a high output voltage. Thus, it can be used as a self-powered vibration sensor with high sensitivity [57,58,59]. Mechanical vibrations are specified by frequency and amplitude. Vibration frequency can be easily detected by most vibration sensors, however, measuring vibration amplitude is more difficult. Measuring one of the vibration physical characteristics such as displacement, velocity, and acceleration with time provides information about the vibration amplitude and frequency. Some TENGs have been developed to measure one of the vibration physical characteristics [57,58,59]. In this article, we summarize the recent progress of utilizing a TENG for energy harvesting and sensing the mechanical vibration of physical objects in the last 10 years. First, the working mechanism and some examples of a TENG, as well as a hybrid TENG for vibration energy scavenging, are presented. Then, measuring mechanical vibrations using a TENG by measuring either vibration displacement or vibration acceleration are reviewed. Finally, some TENGs that can work effectively for both vibration energy harvesting and vibration sensing are described.

## 2. Working Mechanism of a TENG

**Figure 2:**
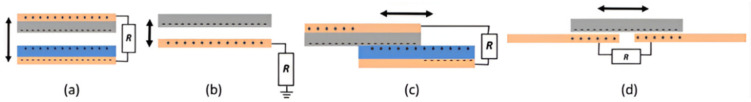
TENG modes: (**a**) vertical contact-separation mode, (**b**) single-electrode mode, (**c**) lateral-sliding mode, and (**d**) freestanding triboelectric-layer mode.

There are four operational modes of a triboelectric nanogenerator (TENG): vertical contact-separation mode, in-plane sliding mode, single-electrode mode, and free-standing triboelectric-layer mode, as shown in Figure 2 [31].

In the vertical contact-separation mode (Figure 2a), two surfaces from dissimilar materials come into contact and then are vertically separated. As a result, oppositely electrostatic charges are generated on the surfaces. Repeated contact-separation and varying the gap between surfaces by mechanical vibrations or another repeated motion generate voltage by electrostatic induction. Both surfaces are attached to electrodes for electrons transfer when a TENG is included in a closed circuit. Thus, mechanical energy is converted to electrical energy [31].

In the single-electrode mode, the triboelectrification is accomplished by contact and separation, as in the contact-separation mode. However, the ground is taken as a reference electrode. This mode is capable of harvesting energy from a freely moving object that is difficult to be attached to a lead or wire (Figure 2b).

The lateral-sliding mode (Figure 2c) has the same structure and starting position as the vertical contact-separation mode. However, triboelectric charges are created on both surfaces by the relative parallel sliding. The lateral polarization along the sliding direction generates triboelectric charges on the contact surfaces, which drive the electrons on the electrode to flow. With repeated sliding and closing, an AC output voltage is generated. This sliding can be in the form of planar motion, disc rotation, or cylindrical rotation [5].

The basic idea of freestanding triboelectric-layer mode (Figure 2d) is that a pair of symmetric electrodes are connected to the external circuit, and the movement of the freestanding triboelectric layer between them allows the current to flow from one electrode to another. The current flow direction depends on the motion direction of the freestanding layer. Usually, the freestanding layer is made from dielectric material, or both electrodes are placed underneath a dielectric layer [60]. The freestanding triboelectric-layer mode can take one of two configurations: a sliding-freestanding TENG or a contact-freestanding TENG [61].

In most of vibration harvesting devices and sensors, the environmental vibrations are transmitted into internal oscillations inside the device via a spring-mass system. Such internal relative oscillations are converted into electricity by electromechanical transduction. For example, the relative oscillation between a permanent magnet and a coil generates EMF by electromagnetic induction, and the relative oscillation between two connected bodies by a piezoelectric element causes deformation/stress on the piezoelectric element, which in response generates electrostatic voltage. However, when triboelectricity is utilized for energy transduction, sort of periodic contact-separation or sliding movements between two surfaces should be implemented. A spring or a kind of elastic/elastomeric mechanism that can provide a restoring force is introduced in the TENG as well, to realize such movements. It is worth mentioning that, when the TENG is utilized for harvesting relative vibration motion between two external surfaces, no mechanical springs are required. The external relative motion can be directly converted into electricity by one of the TENG’s working modes based on the nature of the relative motion. The TENG in that case becomes a non-resonant harvester, by which increasing the input frequency increases the frequency of the electrical output.

## 3. Triboelectric Nanogenerators for Vibration Energy Harvesting

The triboelectric nanogenerator (TENG) is considered as an effective and powerful technology to scavenge electricity from repetitive movements of objects, especially at low frequencies [62]. Mechanical vibration is a kind of repetitive movement that appears in everyday life. TENG technology shows great advantages that are represented in high output voltage, low cost, multiple optional materials, simplicity, and efficient response to low frequency—unsteady motion [63]. Those advantages promote the TENG for motion energy harvesting, especially from random vibrations. Harvesting mechanical vibrations can serve many important applications such as machine and engine monitoring, structure health monitoring, IoT, etc. Thus, researchers turn to take the advantages of the TENG for vibration energy harvesting. A TENG can be used on its own for energy transduction in the harvesting device, or in hybrid with other transduction technologies such as piezoelectric and electromagnetic. Here, we reviewed some of the TENG and hybrid TENG vibration energy harvesters. Table 1 also shows a summary of the reviewed harvesting systems showing their main features and output performance.

### 3.1. TENG-Based Vibration Energy Harvesting

**Figure 3:**
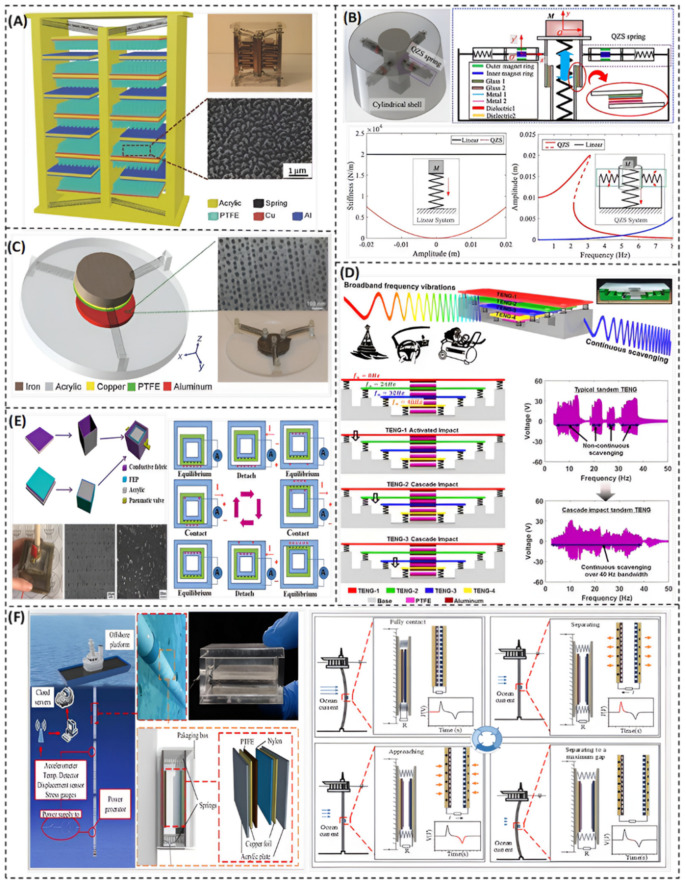
Vibration energy harvesting based on only TENG. (**A**) A 3D stack integrated triboelectric nanogenerator with a wide bandwidth of 36 Hz (Reprinted with permission [64], copyright 2014, John Wiley and Sons). (**B**) An energy harvester based on sliding mode triboelectric nanogenerator with a QZS system to gain an ultra-low stiffness in large displacement region (Reprinted with permission [65], copyright 2021, Elsevier). (**C**) A 3D TENG-based energy harvester that combines two operational TENG modes: vertical contact-separation mode and in-plane sliding mode (Reprinted with permission [66], copyright 2014, John Wiley and Sons). (**D**) A triboelectric nanogenerator with cascade structure based on impact to scavenge a wide range of input frequencies (Reprinted with permission [67], copyright 2019, Scientific Reports). (**E**) A gas-enhanced triboelectric nanogenerator based on fully enclosed structure that replaced the air with SF6 so that the output voltage and output current could be increased by 67% and 120%, respectively (Reprinted with permission [68], copyright 2018, Elsevier). (**F**) A contact-mode TENG to harvest vibrations of marine pipes (Reprinted with permission [69], copyright 2021, MDPI).

Figure 3 shows sample prototypes of recently developed TENG-based vibration energy harvesters. According to the type and style of input motion, a certain mode of the TENG is employed. Yang et al. [64] proposed a 3D stack integrated energy harvester (Figure 3A), which utilizes a multilayer triboelectric nanogenerator. It consists of a 3D acrylic structure with pinned and movable fingers that are installed on a movable acrylic base. The fingers are made from 3 mm acrylic sheets and are placed parallel to each other. The movable base is attached to the structure by eight identical helical springs. The function of those springs is to allow the movement of the movable fingers. This structure is based on the contact-separation TENG mode to harvest motion in a vertical direction.

Thin films of aluminum are affixed to both sides of the pinned fingers to act as an electrode as well as a contact surface. Each side of the movable fingers has a layer of polytetrafluoroethylene (PTFE) with a layer of copper as another electrode. The purpose of the PTFE nanowires, as depicted in the SEM image of Figure 3A, is to increase the triboelectric charges and, thus, the overall electrical output.

The harvester was fabricated and tested through a wide range of vibration frequencies, varying from 2 Hz to 54 Hz. The resonant frequency appears at 40 Hz. Compared to other vibration energy harvesters, it shows a wide bandwidth of 36 Hz in the low frequency range. At the resonance frequency of 40 Hz, the open circuit voltage (Voc) and the short circuit current (Isc) are, respectively, 303 V and 1.14 mA. It has been noticed that with the same number of charges transported back and forth, a faster approaching produces larger current peaks than that during a slower separation. One of the important features of this 3D-TENG harvester is that it can serve different applications based on specific dimensional design. For example, it can be integrated into a ball with arbitrary size and be used to harvest human body kinetic energy while doing some sports such as basketball, football, baseball, etc. Moreover, it can efficiently harvest ocean waves by weaving together a large amount of those self-powered balls [64].

Wang et al. [65] introduced an energy harvester based on the unique idea of combining a quasi-zero-stiffness (QZS) mechanism and a sliding-mode triboelectric nanogenerator, as shown in Figure 3B. The main advantage of the QZS-TENG is to enhance the harvesting performance in the ultra-low frequency region (less than 4 Hz). The QZS-TENG shows a maximal output power of 6 mW at ultra-low frequencies [65]. The main purpose of the QZS mechanism is to create a system with a low resonant frequency (Figure 3B). The stiffness of the linear system is much larger than that of the QZS system. As a result of the low stiffness feature, the vibration response amplitude in the ultra-low frequency region of the linear system is lower than that of the QZS system.

As illustrated in Figure 3B, the QZS spring consists of two magnet rings (with green and blue colors) and two sliding bearings. The sliding bearings are fixed to the QZS spring to reduce the friction between the rod and an axial spring, while its stiffness is neutralized with the pair of magnetic rings. When the QZS-TENG is exposed to an external excitation, the QZS spring will rotate around the hinge, which is fixed onto the cylindrical shell, and the inner magnet ring moves from the equilibrium position in the horizontal direction by a distance. This will result in a thrust force along the axial direction. So, a negative stiffness will be provided by the QZS spring for the QZS-TENG along the vertical direction. Under the platform of mass M, there is a sleeve with four bonded glasses fixed on the frame of the QZS-TENG and four metal electrodes bonded with four dielectrics. Both the metal electrode and the dielectric are bonded on the glass. There are four similar configurations, which are fixed onto the platform, so that a sliding-mode triboelectric nanogenerator is formed, as shown in Figure 3B. This unique idea can be used in several applications of energy harvesting such as environmental and structure health monitoring [65].

Yang et al. [66] developed a multiple direction broadband TENG-based vibration energy harvester (Figure 3C). The developed system has an innovative design which could operate by combining two modes of the TENG: the vertical contact-separation mode and the in-plane sliding mode. This hybrid mode system allows harvesting random vibrations with different directions and with a wide bandwidth of relatively high frequencies. As schematically shown in Figure 3C, this 3D-TENG system consists of an acrylic base, a cylindrical core that lies in the center of the base, and three identical springs separated by an angle of 120° between each other. Those springs are connected to the cylindrical mass and the acrylic supporting base. The symmetrical design of the system allows it to have a constant resonant frequency in in-plane directions. There is a layer of polytetrafluoroethylene (PTFE) thin film pasted to the bottom side of the cylindrical mass as well as a copper electrode that acts as a back electrode. Moreover, there is a thin film of aluminum adhered to the acrylic base that has a dual function of a contact surface and a conductive electrode. The vertical contact-separation mode shows a wide working bandwidth of up to 75 Hz, while the in-plane sliding mode has a relatively lower bandwidth of 14.4 Hz [66]. This 3D-TENG has been tested, and the results were very promising for use in several practical applications such as self-powered sensors, infrastructure monitoring, and charging portable electronics. It has a maximum power density of 1.35 W/m^2^ for the out-of-plane excitations and 1.45 W/m^2^ for the in-plane excitations. These results exhibit the influence of hybridization between the two modes of the TENG.

For unsteady random vibrations, Bhatia et al. [67] proposed a tandem triboelectric nanogenerator with cascade impact structure (CIT-TENG) to continuously scavenge broadband vibrations (Figure 3D). The design concept of this harvester is based on resonance. It consists of four TENGs; each one has a different resonant frequency that enables the harvester to operate at a wide frequency range from 0 to 40 Hz. It can also work and produce remarkable output power away from resonance, which is due to the cascade impact structure of the harvester. Simulation and experimental results show that the impact dynamics of the CIT-TENG enables scavenging energy even under low input accelerations of 0.2–0.5 G m/s^2^. It can also produce a continuous output voltage of 15 V over 40 Hz bandwidth. This CIT-TENG can be utilized in several applications without the need for redesign validation, such as a car dashboard, water waves, and air compressor machines [67].

A gas-enhanced triboelectric nanogenerator based on a fully enclosed structure (Figure 3E) was developed by Lv et al. [68]. They came up with the idea of using SF6 atmosphere instead of air. This increases the output voltage and current by 67% and 120%, respectively, compared to normal atmospheric air. The proposed cubic TENG is composed of two boxes inside each other. The inner box dimensions are 3 × 3 × 3 cm^3^, while the outer box dimensions are 5 × 5 × 5 cm^3^. The outer surfaces are covered with the etched fluorinated ethylene propylene (FEP) film with a thickness of 0.1 mm, except the top and bottom surface of the inner box. Two small springs are attached to the bottom center of the outer box, while the inner box was fixed with a glue on the top of the two springs. The box is completely sealed with a waterproof sealant.

This cubic TENG can harvest vibrational energy from four sides, as the triboelectric material covers the cubic faces. When the box is subjected to mechanical vibrations, the FEP film on the inner box makes contact-separation with the conductive fabric on the outer box. Thus, the two surfaces are positively and negatively charged, and a current is generated through the external circuit by electrostatic induction. The cubic TENG was optimized when filled with SF6, which produced a maximum output voltage of 500 V and a current of 11 µA. Those values were not affected by the ambient humidity.

Some TENG-based vibration energy harvesters are designed for specific applications. For example, Li et al. [69] introduced a contact-separation mode TENG to harvest marine pipe vibrations (Figure 3F). There are many sensors for structural monitoring of marine pipes, which need to be powered. This TENG exploits the vibration of the pipes, which comes from the ocean, to power the sensors. The device is composed of dielectric material films adhered to a simple mass-spring system, to ensure the contact-separation motions of the triboelectric materials [69]. The working cycle of this device has four stages, which occur in sequence: fully contacting, separating, separating to a maximal distance, and approaching. Each stage is shown in Figure 3F. The experimental tests have proven that the electrical output of the TENG device could meet the requirements of the monitoring systems in pipes, since the output power of the TENG has a maximum value of 14 μW with a power density of 5.56 mW/m^2^ at a resistance of 200 MΩ.

### 3.2. Hybrid TENG-Based Vibration Energy Harvesting

**Figure 4:**
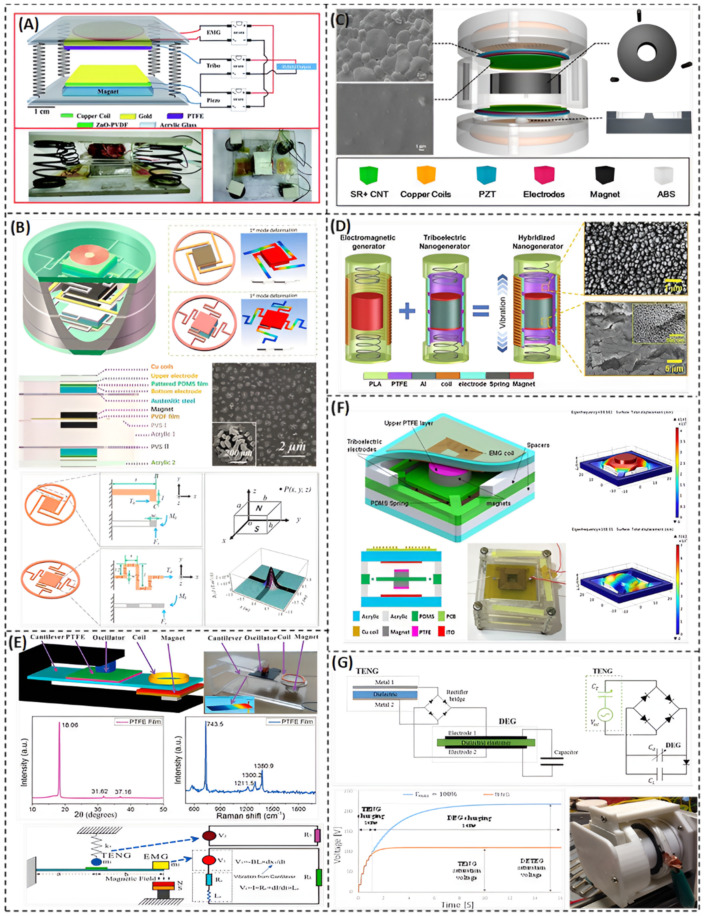
Hybrid TENG-based vibration energy harvesting. (**A**) Vibration energy harvester based on combining different devices in order to increase the efficiency and applicability (Reprinted with permission [70], copyright 2021, Royal Society of Chemistry). (**B**) A piezoelectric–electromagnetic–triboelectric hybrid energy harvester that can scavenge vibrations with frequency ranged from 3.5 Hz to 10 Hz (Reprinted with permission [71], copyright 2017, Elsevier). (**C**) Cylindrical triboelectric–piezoelectric–electromagnetic hybrid nanogenerator that is mainly based on a magnetic levitating structure (Reprinted with permission [72], copyright 2017, Elsevier). (**D**) A hybridized electromagnetic–triboelectric nanogenerator for harvesting human vibrations that are less than 5 Hz (Reprinted with permission [40], copyright 2020, Elsevier). (**E**) An electromagnetic–triboelectric nanogenerator based on multi-impact technique for harvesting wideband frequency vibrations (Reprinted with permission [73], copyright 2017, MDPI). (**F**) Broadband energy harvester utilizing non-linear polymer spring and electromagnetic–triboelectric hybrid harvesting mechanism (Reprinted with permission [74], copyright 2021, Scientific Reports). (**G**) Hybrid dielectric elastomer–triboelectric nanogenerator with enhanced output performance (Reprinted with permission [75], copyright 2021, Elsevier).

The hybridization of a TENG with other transduction mechanisms, such as piezoelectric and electromagnetic, is proposed to improve the output performance. A hybrid generator is usually developed for higher output power, higher power density, higher energy conversion efficiency, wider bandwidth, better environmental adaptability, and/or better applicability. Singh et al. [70] have designed a harvester that utilizes the three transduction techniques to get the most benefit from the size and achieve the highest power density in a cuboid shape (Figure 4A). The harvester can produce an open-circuit output voltage of 192 V because of the hybridization. It uses a ZnO–PVDF film for the combination of the piezoelectric and the triboelectric effects. The harvester design allows the acquisition of the simultaneous output power of each effect from a single vibration direction. It consists of two acrylic bases connected by four identical springs, which allow the excitation of the system in the vertical direction. The vertical excitation creates a contact-separation motion on the TENG as well as pressure on the piezoelectric material, as shown in Figure 4A. Due to the dual nature of the ZnO–PVDF material, the triboelectric effect and the piezoelectric effect have been combined [70]. The electromagnetic harvesting effect is implemented by a copper coil attached to the upper acrylic base, and a permanent magnet fixed to the other base. When the two bases approach each other, the magnetic flux changes across the coil and generates electricity by electromagnetic induction (EMG). The performance of this hybrid generator is checked with some applications just like lighting several LEDs connected in series, powering a digital calculator, and a screw gauge meter [70].

The hybrid generators can have the advantage of broadband performance, which can work effectively at a wide range of input frequencies. He et al. [71] introduced a piezoelectric–electromagnetic–triboelectric (PET) hybrid generator (Figure 4B), which can efficiently scavenge vibrational energy with frequency ranging from 5 to 40 Hz. It has an output voltage of 12.6 V at 20 Hz and 13.3 V at 24 Hz [71]. The structure of the generator is in a form of cylindrical acrylic case with three layers of hybrid energy harvesters, as shown schematically in Figure 4B. The middle layer is a piezoelectric–electromagnetic hybrid harvester. It has a unique structure, which consists of four L-shaped PET beams with flexible PVDF piezo film pasted on them, two permanent magnets, and other two magnetic coils. This structure is called the picking up vibration structure (PVS) I. The top and bottom layers of the harvester are the same and are placed symmetrically. Each one is a triboelectric energy harvester, which is based on the picking up vibration structure (PVS) II. (PVS) II includes four PET folded beams. Each harvester of the three previously is considered as a lumped spring-mass-damper system. Each system has a resonant frequency according to its structure. Both structures have two close resonant frequencies. In addition, the excitation of any of these harvester causes the other harvester to be excited, due to them hitting against each other. This allows the cylindrical PET energy harvester to operate at a very low and wide range of frequencies [71].

He et al. [72] presented a compact hybrid generator (Figure 4C) with high power density that is able to work efficiently at low frequencies. The proposed harvester has a cylindrical compact design with dimensions of 48 mm × 27 mm. It has a light weight due to the low-density materials and is capable of scavenging vibrational energy under 20 Hz. This low frequency operating range is a result of combining the three mechanisms of transduction: piezoelectric, triboelectric, and electromagnetic. The design of this PET harvester is so simple and has an innovative idea for the mechanism of movement. It is all about the movable core, which is a permanent magnetic core levitating in the middle of three cylindrical magnets in a triangle-shape distribution. According to the layout design of the harvester (Figure 4C), the levitated annular magnet has three roles in generating electricity from the PEG, EMG, and TENG. The first role is generating electricity from the piezoelectric effect, due to the impact between the PZT sheets and PEG units in the harvester. The second one is changing the magnetic flux in the two copper coils existing in the top and bottom of the system. This results in generating electricity by electromagnetic induction. The third role is from there being a triboelectric electrode in the TENG unit, which delivers electrons between the two rubber layers at the top and bottom of the harvester during the up and down vertical oscillation [72]. The presented harvester can generate 78.4 µW output power due to the triboelectric nanogenerator (TENG), 36 mW and 38.4 mW due to the electromagnetic generators in the top and bottom of the system, respectively, and 122 mW and 105 mW from the piezoelectric generator in the top and bottom of the harvester, respectively [72].

Energy harvesting of ultra-low frequency vibrations such as human walking can serve important applications. It can be a sustainable power source for portable electronics and wearable sensors [45,76,77]. Rahman et al. [40] invented a miniaturized freestanding kinetic-impact-based hybridized nanogenerator (MFKI-HNG) that is capable of harvesting energy from human body motion in the ultra-low frequency range (Figure 4D). This MFKI-HNG is designed to generate electricity simultaneously from an electromagnetic generator (EMG) and a triboelectric nanogenerator (TENG) in a simple way. The system configuration is like a battery, as they both have almost the same shape and size. The harvester consists of a cylindrical aluminum mass oscillating in a cylindrical tube, and two cylindrical magnets are fixed to the top and the bottom of the mass. Moreover, there are two identical springs; each has a fixed end to the end of the tube, while the other end is free. The copper coil is wound over the outer surface of the tube, where the magnetic flux changes due to the magnets’ movement up and down and, consequently, generates electricity by electromagnetic induction. A layer of triboelectric material partially covers the internal surface of the tube, which creates, with the cylindrical mass, a sliding mode TENG and generates electricity by the triboelectric effect [40]. Figure 4D shows the results of the harvester output. By optimizing the parameters, it can generate an output voltage of 18 V under 6 Hz. It also generates a maximum power of 102.29 mW and a power density of 3.67 mW/cm^3^. This power value can be sufficient for powering smartphones and smartwatches.

Due to the convenience and effectiveness of a device like that invented by Rahman et al. [40], a few hybrid generators were presented with the same concept but with different designs. One was introduced by Seol et al. [78] in 2016. A magnetic-based spring was utilized instead of a mechanical one. Two permanent magnets were fixed to the ends of the tube, which provides repulsive forces, and two copper coils were wound over the tube. The operation range of frequencies was under 10 Hz and the output power from the EMG and the TENG was 128 W/Kg m^3^ and 130 W/Kg m^3^, respectively. Another energy harvester was that presented by Salauddin et al. [79] in 2017. It was composed of two rectangular-shaped cylinders inside each other with a magnetic stiffness between them and copper coils. The fabricated hybrid energy harvester gave an output power of 10.07 mW and an output current of 3.74 mA at a resonant frequency of 4.5 Hz, which corresponded to a 344 W/m^3^ volume power density.

Zhu et al. [73] presented a hybrid broadband TENG—EMG device. As shown in Figure 4E, the device consists of a PTFE cantilever, where the TENG and EMG are placed at the middle and the end of the cantilever, respectively. It has a wide operating low frequency bandwidth (10–45 Hz) due to the multi-impact approach, which introduces a nonlinearity in the system. It also has a small-size device, which could be suitable for powering small electronics just like smartphones and smartwatches. From the results shown in Figure 4E, the proposed harvester can generate an average power from the TENG and the EMG up to 30 µW/m^2^ and 53 µW/m^2^, respectively [73].

A way of combining non-linear stiffening effect with the dual-way energy harvesting (TENG and EMG) is introduced by Gupta et al. [74]. They presented a TENG—EMG hybrid generator with a wide bandwidth of 23–68 Hz over a wide range of input acceleration (0.1–2.0g) (Figure 4F). There are two working modes of this device, as shock and resonant. Harvesting energy in resonant mode is achieved at 82 Hz with output voltage of 20 mV and 55 mV from the TENG and EMG, respectively. Whereas energy harvesting in the non-linear mode or hand tapping mode reaches a maximum power density of 4.8 µW/cm^3^ from the TENG and 6 µW/cm^3^ from the EMG.

Hybridization between TENG technology and the other technologies such as EMG and PEG is proven to be an innovative strategy to boost the output power, increase the operating bandwidth, and efficiently harvest low and ultra-low frequency vibrations. Though, there are other transduction technologies that can benefit from coupling with a TENG, such as dielectric elastomers (DE). Haroun and Lee [75] developed a DE—TENG hybrid generator, namely DETEG (Figure 4G). A dielectric elastomer has the advantages of stretch ability, high energy density, and the ability to match low frequency motion. It deforms with nonlinear stiffening behavior, which can work as a nonlinear self-spring and be utilized for broadband vibration energy harvesting. However, a dielectric elastomer cannot convert mechanical energy to electrical energy on its own. It needs a prime voltage or an external source of prime electrical charges. It converts mechanical energy to electrical energy by amplifying the prime voltage to higher voltage levels. Therefore, tethering a TENG with a DEG can provide the necessary prime voltage to the DEG, which in turn is amplified by the external input mechanical energy. The hybrid DETEG system was introduced to gain use of the DEG advantages mentioned before, for portable applications. In addition, the DE membrane has an electric field breakdown strength larger than air. A higher voltage can be sustained across the DE membrane. Thus, DETEG can be more convenient with high voltage applications such as the powering of dielectric elastomer actuators (DEA) and the self-powering of electrospinning systems.

## 4. Triboelectric Nanogenerators for Vibration Measurements

Vibration measurement is a key component of machine-condition monitoring, which enables early detection of machine damages, faults, and abnormal stoppings. A TENG can convert mechanical motion in the form of contact-separation or sliding between two surfaces into electrical output with high voltage. Therefore, a TENG can be used as a vibration sensor with high sensitivity, by converting vibration motion into one of a TENG’s modes of contact. A TENG for vibration sensing uses the same concept as a TENG for vibration energy harvesting. However, other important features are considered in case of vibration sensing such as sensitivity, resolution, measuring range, and linearity. Vibration motion is mainly defined by vibration amplitude and frequency. Vibration frequency can be easily detected by most vibration sensors, but the amplitude is more difficult. Measuring one of the vibration physical characteristics such as displacement velocity or acceleration with time provides information about vibration amplitude and frequency. Some TENGs have been developed to measure either vibration displacement or vibration acceleration, which are reviewed in this work.

### 4.1. Triboelectric Sensors for Displacement Measurements

**Figure 5:**
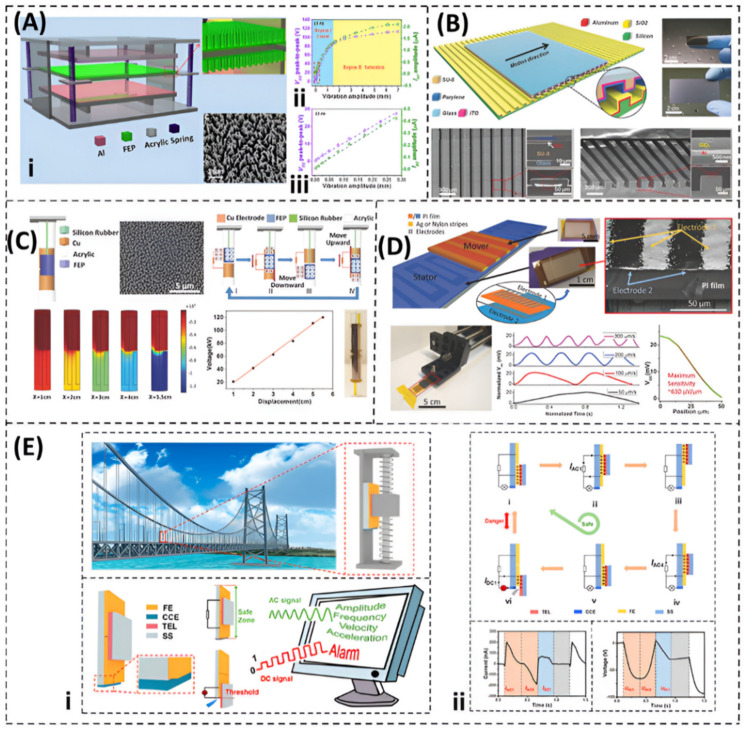
Triboelectric sensors for vibration-displacement measurements. (**A**) A contact-separation-enabled freestanding TENG sensor for measuring vibration displacement amplitude, (Reprinted with permission [80], copyright 2014, American Chemical Society). (**B**) A single dimensional displacement and speed sensor technology based on two layers of micro-grated triboelectric layers (Reprinted with permission [81], copyright 2013, John Wiley and Sons). (**C**) An integrated self-powered dynamic displacement monitoring system for structural health monitoring (Reprinted with permission [82], copyright 2017, John Wiley and Sons). (**D**) An aerosol jet printing (AJP) that is used to create triboelectric sensors from different materials, including polymers, which may be directly printed into finely detailed grating structures for increased sensitivity to displacement and speed of motion (Reprinted with permission [83], copyright 2018, John Wiley and Sons). (**E**) A self-powered vibration sensing system that can detect vibration properties in real time, continuously using a dual-mode triboelectric nanogenerator that can create either alternating current (AC) or direct current (DC) within various operation zones (Reprinted with permission [84], copyright 2020, American Chemical Society).

Vibration displacement is an important characteristic that represents the strength of mechanical vibrations. Some TENGs are developed to measure only the displacement amplitude, while others measure the vibration displacement over time, which can provide information about both vibration amplitude and frequency. Wang et al. [80] proposed a contact-separation-enabled freestanding TENG (CF-TENG) for self-powered displacement amplitude measurements. The configuration of the system is schematically shown in Figure 5A. Two Al-deposited acrylic plates are supported in a parallel setup with a gap of 2 cm, which act as the stationary electrodes. An acrylic sheet is placed between them and supported with eight springs through its corners, which acts as a vibration resonator. Two 50-μm fluorinated ethylene propylene (FEP) films are laminated onto the two sides of the acrylic sheet. They are purposely chosen as the freestanding triboelectric layers due to their distinctively opposite triboelectric polarity with Al. When the system is excited with input vibrations, the resonating acrylic sheet allows the two triboelectric layers to alternatively reach the electrodes. When the vibration amplitude is high, the generation of triboelectric charge will occur due to contact between the FEP layers.

The resonator plate reaches the largest vibration distance at resonance. The resonant frequency in this system is around 15 Hz, at which the best sensitivity of the system can be found. When the system works at 15 Hz, as the amplitude increases, the open circuit voltage and the short circuit current increase almost linearly, until the amplitude reaches 1.5 mm. This is called region I, as depicted by Figure 5A(ii). After 1.5 mm, the acrylic plate starts to impact with the electrodes, which is called region II. Figure 5A(iii) shows a linear relationship between the open-circuit voltage and the vibration amplitude over a wide range of displacements. This linearity indicates the performance enhancement of the CF-TENG over simple contact-mode TENGs that generate remarkable signals only within narrow limits of separation distance. It also gives an advantage to the CF-TENG when utilized as an energy harvester, since it would have the capability to harvest large amplitude vibrations. This sensor can detect small displacement amplitudes down to 3.5 µm at the resonant frequency and up to 15 mm at a frequency of 21 Hz. This refers to a good sensitivity of the sensor.

Wang et al. [81] proposed a nanometer-resolution self-powered static and dynamic sensor, based on micro-grated triboelectrification, which consists of a pair of micro-grating structures with relative motion between them that leads to the periodic separation of two grates that have opposite charges, as shown in Figure 5B. The sensor can detect the vibration displacement by the open circuit voltage and the short circuit current generated by the sensor. The motion range of this sensor is 200 μm, and the resolution is 173 nm, with a linearity error of 0.02%. The displacement can be calculated by identifying the number of peaks in the output signal. One peak is an indication of 200 μm displacement. When the sensor is placed on a vibration generator and the number of zero-crossings of the output voltage are counted, each of which represents a 100 μm step of displacement, then the real-time displacement can be derived.

Yu et al. [82] presented a self-powered dynamic displacement monitoring system, as illustrated in Figure 5C. It consists of an outer tube made of acrylic with an inertial mass inside, which is suspended by a stretchable silicone rubber wire. Two nylon films coated with copper are glued to the inner surface of the tube, which provide an optimized gap of 2 mm inside the tube. The fluorinated ethylene propylene (FEP) film, as the freestanding triboelectric layer, is coated on the cylindrical mass that has a diameter of 15 mm and weight of 32 g. The external input motion causes a relative motion between the mass and the tube. The resulted open circuit voltage shows an acceptable linearity with the displacement of the inertial mass. The natural frequency of the proposed sensor is important, which can be determined by selecting the appropriate size and material parameters, such as wire elastic modulus, wire length, and cylinder diameter. The natural frequency of the proposed prototype is designed to be 434.8 Hz. In this sensor, the dynamic displacement monitoring system automatically sends an alarm signal when the displacement amplitude exceeds a pre-set limit. The testing results of the sensor show that it behaves accurately below 5 Hz, compared to a traditional piezoelectric accelerometer. Moreover, it has a high sensitivity of 0.391 V s^2^/m when it operates at 3 Hz.

Jing et al. [83] use the aerosol jet printing (AJP) technique to develop novel triboelectric sensors for high-resolution motion sensing, including displacement, as shown in Figure 5D. This is done by direct and rapid printing of both metallic and polymeric inks into the desired finely grated structures. The fabrication of an ultra-fine grated and patterned interdigitated electrode structure is done by printing silver and polyimide inks onto flexible polyimide substrates. These make up the main functional parts for the mover and the stator of the sensor. The stator contains a pair of interdigitated electrodes at a pitch of 50 μm. The centers of the comb electrodes on either side are displaced by half a pitch to form a pair of interdigitated electrodes. For the mover, a grated pattern with a period of 100 μm was printed on polyimide film with Ag ink. Material selection of triboelectric layers is critical when designing this system. So, in this work, optimization was done by printing three ink movers with different combinations of metals as follow: Ag on Kapton, PVDF-TrFE on Kapton, Kapton on nylon, Kapton on Teflon, and Kapton on aluminum foil. When the layers are tested under the same condition, the highest output voltage is acquired from the Ag–Kapton combination, which is an interesting result. This sensor has a high resolution of up to 50 µm, with a maximum sensitivity of around 630 mV/µm. Aerosol jet printing shows a high resolution, a low prototyping time, wide material options, and a large scale.

To achieve a real time monitoring of structures and predict vibrations behavior with a warning system, a self-powered vibration monitoring system is introduced by Li et al. [84], as shown in Figure 5E. The proposed system can detect vibration characteristics (e.g., displacement amplitude, frequency, and acceleration) as well as detect the working state of the construction with an alarm system, when reaching the safety threshold. The configuration of the AC/DC-TENG consists of a stator and a mover, as shown in Figure 5E. The stator is made of two friction electrodes, a charge-collecting electrode, and an acrylic layer that acts as a frame. The two friction electrodes are equal in size (20 mm × 20 mm), and are pasted side by side on the acrylic layer with a gap of 0.5 mm. The collecting electrode is put on the bottom edge of the acrylic layer. The slider (20 mm × 20 mm) is attached to a fluorinated ethylene film as a triboelectric layer. When the mover oscillates between the two friction electrodes without exceeding the edge of the acrylic layer (the safe zone), an AC signal is generated by triboelectrification and electrostatic induction, and, hence, the vibration characteristic is obtained.

The working mechanism of the device is shown in Figure 5E(ii). Initially, the mover is fully overlapped with the friction electrode. In the safe region, the case (vi) will not occur. If part of the slider moves off the edge of the friction electrode, the case (vi) occurred. Due to the high electrostatic field between the collecting charge electrode and the negatively charged friction electrodes surface, air breakdown occurs in the gap, causing a DC in the external circuit. This was confirmed by the same author in previous research [85]. In the sage zone, the open circuit voltage is proportional to vibration displacement amplitude. The device was tested for more than 10,000 cycles, to prove a high stability. The system also shows a linear behavior in both the safe and danger zones.

### 4.2. Triboelectric Sensors for Acceleration Measurements

Acceleration sensor is an important tool in vibration condition monitoring and others. It plays an essential role in many applications such as GPS devices, biomedical devices, artificial intelligence products, automotive, environmental monitoring, and equipment monitoring. In recent years, the triboelectric nanogenerator has been developed as a promising technology for acceleration sensing [86]. The most important features of the reviewed TENG displacement and acceleration sensors are summarized in Table 2.

**Figure 6:**
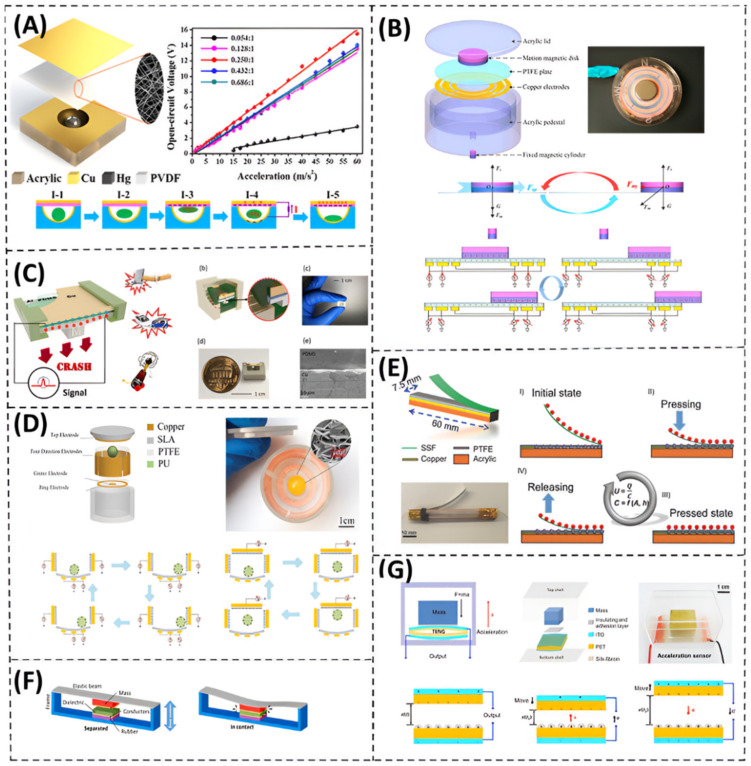
Triboelectric sensors for acceleration measurements. (**A**) Self-powered and extremely sensitive triboelectric acceleration sensor that is made of a liquid metal mercury droplet and a nanofiber-networked polyvinylidene fluoride film (Reprinted with permission [87], copyright 2017, American Chemical Society). (**B**) Self-powered multifunctional triboelectric motion sensor (MFMS), which is capable of concurrently sensing motion characteristics such as direction, speed, and acceleration of linear and rotating motions (Reprinted with permission [88], copyright 2018, American Chemical Society). (**C**) Self-powered triboelectric high-g acceleration sensor (Reprinted with permission [89], copyright 2017, Elsevier). (**D**) Self-powered multifunctional triboelectric sensor based on PTFE and PU for vibration and motion sensing (Reprinted with permission [90], copyright 2020, John Wiley and Sons). (**E**) Multifunction sensor based on elastic-beam triboelectric nanogenerator. It consists of copper and polytetrafluoroethylene layers that are glued on an acrylic plate (Reprinted with permission [91], copyright 2018, John Wiley and Sons). (**F**) Inertial triboelectric motion sensor with optimized parameters (Reprinted with permission [92], copyright 2021, Elsevier). (**G**) Self-powered and high-sensitivity triboelectric acceleration sensor. It is composed of transparent outer shell and inner mass and silk fibroin patches (Reprinted with permission [93], copyright 2019, Elsevier).

Based on a liquid metal triboelectric nanogenerator, a self-powered acceleration sensor is introduced by Zhang et al. [87], as shown in Figure 6A. The sensor was tested for over 200,000 cycles. It shows a high sensitivity of 26 Vs/m^2^ and a wide working range from 0 to 60 m/s^2^, with excellent stability and durability. The high stability and durability are achieved due to the usage of liquid metal mercury. The sensor also shows a good linear relationship between the measured acceleration, and the output voltage and current. The graph in Figure 6A shows the effect of different volume ratios of the mercury droplet and acrylic cavity on the measured accelerations.

This self-powered acceleration sensor consists of an internal mercury droplet and external acrylic shell. In the acrylic face, there is a hemisphere cavity with a deposited copper film that acts as an electrode. The nonstick mercury droplet was put in the cavity as a free moving electrode, and the face of the cavity is made from a copper layer coated with nn-PVDF which acts as a TENG layer. The working mechanism of the self-powered acceleration sensor is shown in Figure 6A. At the initial state, the mercury sticks on the bottom of the cavity with no electric charge on the surface of the nn-PVDF layer. When the sensor moves up, the mercury will contact the nn-PVDF layer. Since, they are different in their triboelectric polarity, positive and negative charges are generated on the mercury and on the PVDF layer surface, respectively. When there is no contact between the mercury and the PVDF layer while the sensor is moving down, the equilibration of the electric field is changed, and then an electric difference is created between the electrodes. The electrons will flow from the top electrode to the bottom electrode until the equilibrium of the collected charges.

Wu et al. [88] presented a self-powered multifunctional motion sensor that can detect acceleration of linear and rotary motions, as shown in Figure 6B. The dimension of the device are 40 mm diameter and 19 mm height. The body is made from acrylic. The TENG is composed of a magnetic disk and PTFE as a friction layer, with copper electrodes stuck onto it. There are an inner circle electrode, an outer circle electrode, and arc electrodes between them. By increasing the number of electrodes, the sensitivity increases. For the sake of design, the number of electrodes in this device are stated as four. The four arc electrodes are referring to as north, east, south, and west.

Measuring the linear acceleration is experimentally checked. When the magnetic disk moves linearly in any direction, crossing the inner and outer circle electrodes, the electrostatic potential of the two circle electrodes reach each peak. The voltage waveforms of the inner and outer circle electrodes show the relationship between the voltage and the motion position. When the magnetic disk is at the center, the open-circuit voltages of the two electrodes equal 0 V. When the magnetic disk is moving far from the inner circle electrode, its open-circuit voltage will reach a certain value. After that, with the magnetic disk moving away from the inner circle electrode and toward the outer circle electrode, the open-circuit voltage of the inner circle electrode will reach a peak value. To determine the acceleration, the time is recorded, and the derivation of its value can be done.

In Figure 6C, a self-powered high-g acceleration sensor based on a triboelectric nanogenerator is proposed for the first time [89]. It has a volume of (14 × 14 × 8) mm^3^, a measuring range of up to 1.8 × 10^4^ g, and sensitivity of 1.8 mV/g with a good linearity. The structure of this sensor is shown in Figure 6C. The positive electrode is made of aluminum foil in a beam shape having hinged weight. The negative electrode is made of a PDMS film that is bonded to the face of the copper plate. When there is no high-g acceleration, a contact between the positive and negative electrodes will occur and there is no voltage output. As high-g acceleration occurs, the aluminum beam will deform away from the positive electrode due to the hinged mass. When the deformation reaches the maximum, a potential difference between positive and negative electrodes occurs and reaches its peak due to the distance between charges. After the impact of acceleration, the deformation of the aluminum electrode is restored, which, in turn, causes the voltage to drop back to zero.

A multifunction sensor based on an elastic-beam triboelectric nanogenerator is introduced [90]. As shown in Figure 6D, the sensor consists of a copper (Cu) layer and a polytetrafluoroethylene layer. Both are glued to an acrylic plate. One end of an arc-SSF is fixed on the acrylic plate that holds the copper and the polytetrafluoroethylene layers. The working principle of the sensor is as follows: by contact-separation between the SSF and the PTFE that is caused by external force, the SSF gains a positive charge and the PTFE gains a negative charge. These charges stay on both layers due to the open-circuit state and the dielectric properties of the PTFE layer. Increasing the pressing would not change the amount of charge, but it changes the capacitance, which is related to the contacting area and the separation distance. At the full pressing, the maximum contact area and the minimum separated distance occur. When releasing, the SSF will return to the initial position to complete the cycle. This sensor can be used as a force sensor, acceleration sensor, sensitive scale, and intelligent keyboard with good performance with sensitivity of 30–900 V/N [90].

Another self-powered multifunctional triboelectric sensor based on the PTFE and PU for vibration and motion sensing is proposed [91]. It is made up of a hollow cylindrical structure with a movable cover and a resin frame (Figure 6E). Seven copper electrodes with 0.1 mm thickness are stuck to the inner walls of the frame. One electrode is placed on the top cover. A center electrode and a ring electrode are placed on the bottom, and four directional electrodes are attached to the side wall of the cylinder. All electrodes are coated with the PTFE layer. A PU ball is inserted in the inner hallow cylinder. The working flow of the sensor (Figure 6E) appears in two working modes. The sensor can achieve multiple sensing functions, due to the different combinations of the ball and the electrodes. The sensor can sense speed, acceleration, and direction.

Adly et al. [92] presented a study on the modeling and optimization of an inertial triboelectric motion sensor. The proposed sensor consists of a U-shaped frame with an elastic beam mounted on its ends (Figure 6F). A counterweight is attached to the beam, with aluminum layer pasted on it as a triboelectric layer. The other triboelectric layer is made of Teflon and is fixed on the bottom of the U-shaped frame, with a rubber layer in between. This device is based on the contact-separation mode between the aluminum layer and the dielectric Teflon layer. As shown in Figure 6F, the system is selected to have a resonant frequency of 30 Hz based on an optimization, and it has been tested under different accelerations ranging from 0.4 g to 1.2 g. The focus of this work is to optimize all the device parameters.

Liu et al. [93] came up with an idea for a self-powered and high-sensitivity TENG acceleration sensor. It is composed of a proof mass attached to an arch-shaped triboelectric layer (Figure 6G). The sensor has a transparent outer shell with the mass inside and silk fibroin patches. The arch layer supports the mass. The working principle of the sensor is based on a contact-separation mode TENG. It has two working phases: when the acceleration is upwards, and when the acceleration is downwards. For upwards acceleration, the proof mass tends to move downwards, forcing the triboelectric arch layers to be in full contact. The electron charges will then transfer from the ITO/PET/SF layer to the ITO/PET layer and meet electrostatic equilibrium. For downward acceleration, the mass will then separate the triboelectric layers, and a potential drop will occur, forcing electrical signal to be generated through the two electrodes. This TENG is highly recommended to serve as wearable devices with an alarm and vibration monitoring systems, which require high sensitivity. It exhibits a high sensitivity of 20.4 V/(m/s^2^) and a power density of 371.8 mW/m^2^.

## 5. TENGs for Both Vibration Energy Harvesting and Vibration Sensing

**Figure 7:**
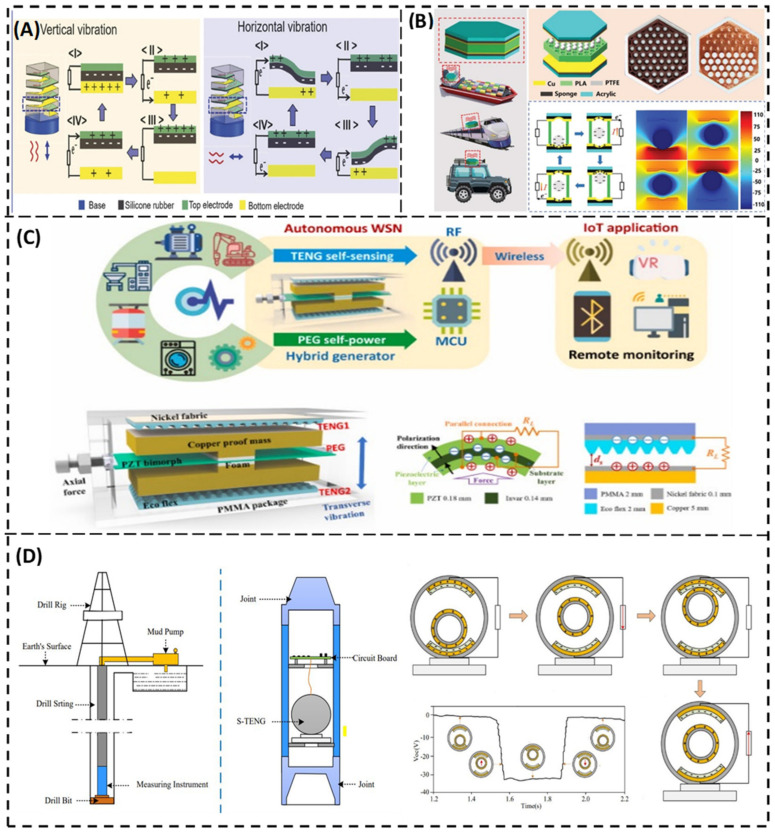
TENGs that can work as energy harvesters as well as self-powered sensors. (**A**) Soft and resilient spring-based triboelectric nanogenerator that scavenges vibrational energy from horizontal and vertical excitations (Reprinted with permission [38], copyright 2019, Advanced Energy Materials). (**B**) Honeycomb-structure vibration sensor for monitoring motors conditions (Reprinted with permission [94], copyright 2019, Advanced Energy Materials). (**C**) Hybridized triboelectric and piezoelectric nanogenerator with a tunable resonant frequency (Reprinted with permission [95], copyright 2020, Nano Energy). (**D**) The structure and mechanism of spherical triboelectric nanogenerator, which is used in drilling process (Reprinted with permission [36], copyright 2020, MDPI).

Some TENGs can work effectively as self-powered vibration sensors as well as energy harvesters. Their designs are suitable for certain sensing applications with sufficient sensitivity. In addition, they have an enhanced output power, which allows them to work effectively as energy harvesters. One example is that presented by Xu et al. [38]. They proposed a bidirectional spring-based triboelectric nanogenerator (S-TENG), as shown in Figure 7A. The spring-based TENG was previously presented, but it can benefit from motion in a single direction. Benefiting from motion in more than one direction at the same time remains a big challenge. Xu et al. succeeded in integrating a spring into a TENG and gaining use of two-directional vibration modes, which come from its bendable property. It has great potential to serve as a self-powered active sensor for acceleration and in harvesting arbitrary vibrational energy generated from buildings, ocean waves, wind, etc. As shown in Figure 7A, the helical structure of the S-TENG is composed of two elastomeric conductive layers, which serve as electrodes. With the aid of wire spring and silicone rubber and through external vibration triggering, both vertical and horizontal vibrations will decrease the adjacent distance between the helical coil, forming a contact-separation mode in the TENG. The S-TENG shows great performance under vertical and horizontal resonant frequencies of 16 Hz and 8.5 Hz, respectively. The average maximum power density was found to be 240 and 45 mW/m^2^, respectively.

Another example of enhancing sensing and harvesting performance is presented by Xiao et al. [94]. They proposed a honeycomb-structure-inspired triboelectric nanogenerator (HIS-TENG). It is composed of two flexible copper electrodes and an intermediate honeycomb frame, which is fabricated using 3D printing (Figure 7B). The overall structure is then framed by two acrylic plates. Each groove in the honeycomb structure, has a poly tetrafluoroethylene ball, which acts as the triboelectric layer. Compared to the square grid structure, the honeycomb structure increases the maximum power density by 43.2% and has an advantage of strong frequency adaptability compared to a spring-assisted TENG. By applying this HIS-TENG on the block of a diesel engine of a real sailing ship, it was found that the HIS-TENG can work as an effective self-powered sensor used for monitoring engine condition; in addition, it has a remarkable performance and great potential in vibration energy harvesting.

The working mechanism of the HIS-TENG simply depends on the motion of the small ball inside each groove. When the PTFE ball is in contact with the lower copper film, electrons are transferred from the bottom electrode to the PTFE ball, which in return will achieve positive and negative triboelectric charges on the PTFE ball and the surface of the electrode layer. As the structure is subjected to any external source of vibrations, the PTFE ball will move upward and separate from the bottom electrode, while hitting the upper one. During this separation, electrons are transfered from the bottom electrode to the top electrode, resulting in a current and charged signals transfer to be generated in the external circuit. This work proved that the HIS-TENG exhibited a robust performance, and it can serve as a self-powered engine condition monitor and may have a great impact on machine monitoring in the future [94].

We all know that autonomous wireless sensor nodes play an important role in the field of industry and Internet of things. That’s why a self-sustained power supply for sensors is highly recommended. Wang et al. [95] proposed a self-sustained autonomous wireless sensing based on a hybridized TENG and PEG vibration mechanism. Their proposed mechanism is shown in Figure 7C. It has a broadband behavior and tunable resonant frequency, which can be applied for various ranges of vibration frequencies. What makes the hybridization of a TENG and a PEG so perfect in this device is that the PEG generates a high current and is more suitable to act as energy harvester, while the TENG has a high voltage output and is preferred to be a self-powered accelerometer, as it has good sensing capability. This hybrid WSN is composed of one PEG and two TENGs. There is a Lead Zicronate Titanate (PZT) bimorph, which is hinged-hinged mounted. There are two T-shaped copper proof masses, in which they are bonded with the PZT bimorph through a piece of foam. This piece of foam will reduce the concentrated stress. The bolt and nut fixed at the side of the outer package give an axial force to tune the resonant frequency of the PZT bimorph. The two TENGs work as energy harvesters as well as act as stoppers for PEG overload, protecting it. The triboelectric positive electrode is a nickel fiber, while ecoflex with a pyramidal synaptic array is selected to act as a negative electrode. This material is selected for its soft stiffness, which ensures sufficient contact in the contact-separation mode and generates more triboelectric charges. Its working mechanism depends on the strength of the vibration excitation. As with stronger vibration and acceleration, more output voltage and current would be generated. When the outer package receives an ambient vibration, the proof mass will force the bimorph PZT to strain, and this will generate electricity to power the acceleration sensor. When the proof mass hits the nickel fabric and full contact occurs, triboelectric charges are transferred to the ecoflex material through the contact-separation mode. This separation is translated to an electric signal through the triboelectrification process. This mechanism can be applied on a train cabin to monitor the conditions and overload status through the vibrations exerted by the moving cabins. It produces an output power of 6.5 mW at 25 Hz and 1.0 g [95].

Another TENG for vibration measurements and energy harvesting from mechanical drilling was invented by Wu et al. [36]. They proposed a spherical triboelectric nanogenerator (S-TENG) for scavenging and measuring mechanical vibrations during the traditional downhole drilling process (Figure 7D), which can be used for exploitation applications such as the exploitation of petroleum, gas, or other mineral resources. Drill string vibrations inevitably occur during any drilling process. With excessive vibrations, the drilling tool would be damaged, and accidents may occur. To avoid that hazard, there must be a method for real-time vibration measurements of the downhole drill string. The traditional methods used for drilling vibrations measurement (using cables and batteries) increase the drilling cost and decrease the drilling efficiency. However, the S-TENG brings hope for solving these problems. The S-TENG consists of a fixed ball, a freely moveable ball, and a supporting seat. The fixed ball is attached to the supporting seat and the moveable ball is embedded inside the fixed ball, as shown in Figure 7D. On the upper and lower sections of the fixed ball there are copper, and polytetrafluoroethylene (PTFE) pasted layers. The Cu serves as the electrode, while the PTFE is used as the friction layer for generating electric charges. When the drill string vibrates, the moveable ball moves up and down relative to the axial vibration of the drill string, generating charges due to the triboelectric effect and electrostatic induction. When the moveable ball separates from the lower seat/lower friction layer, electrons flow through the external load and form electric current. When the moveable ball contacts the upper friction layer (negatively charged) and separates again, electrons move through the external load in reverse, and a reverse current is generated. This S-TENG generates an electrical output of 70 V, 33 µA, and 10.9 nW at a maximum frequency value of 8 Hz [36].

## 6. Conclusions

Mechanical vibrations are an abundant source of motion in the surrounding environment. They appear almost everywhere: in manufacturing machines, cars, trains, household equipment, turbines, etc. That is why researchers have thought about mechanical vibrations as a sustainable energy source for wireless sensors associated with IoT and sensor network applications. Other renewable energy sources are employed such as solar and wind energies. Nevertheless, mechanical vibrations are preferred when the sensors are placed in deep and dark environments. In the past few decades, EMGs and PEGs have shown success in vibration energy harvesting for a wide range of applications. However, the prosperous development of the TENG shows its great potential for vibration scavenging. The TENG has a high energy conversion efficiency, low cost, simple fabrication method, and the ability to match low frequency vibrations. For vibration to electrical energy conversion, the TENG utilizes a spring-mass system or other elastomeric mechanisms that can provide a restoring force. Such a restoring force mechanism guarantees a sustained internal oscillation with the input vibration, which is converted into electricity through one or more of the TENG modes of contact. TENG-based energy harvesters can rely on only triboelectric as a mechanism of energy transduction or a hybrid with other transduction technologies such as electromagnetic and piezoelectric. Hybridization of the TENG is proposed to increase the system power density, the output power, the operating bandwidth, and/or provide a better environmental adaptability and applicability. Some recent developed TENGs and hybrid TENGs are reviewed in this paper. The development of the TENG for vibration scavenge depends on increasing the system output power, widening the frequency bandwidth, having better applicability with certain applications, and/or matching lower frequencies. Those are achieved by utilizing innovative system designs, and, in some cases, by boosting the triboelectrification performance [68]. There is still a potential for the future development of the TENG for vibration scavenging. Some vibration mechanisms, such as a bistable compliant mechanism [96], and variable stiffness [97] mechanisms can be employed with the TENG to enhance its harvesting performance.

The effectiveness of the TENG for vibration energy harvesting encourages researchers to consider it as a self-powered vibration sensor. The TENG converts a mechanical vibration trigger into an electrical signal. So, it can sense vibrations directly without applying a power unit to the device. The TENG shows many advantages when utilized as a vibration sensor, such as high output voltage, high efficiency, low cost, stability, and robustness. Due to the four different TENG modes of contact, it has also great design adaptability and different design possibilities. One of the most important applications of vibration measurement is the machine condition monitoring. Machine condition monitoring helps to detect early signs of deterioration, and it is the core element of machine predictive maintenance. In this work, the recent progress in the development of a TENG for vibration measurements is reviewed as well. Vibration measurements can be achieved by measuring either vibration displacement or vibration acceleration. Unlike a TENG for vibration energy harvesting, other criteria are considered when a TENG is utilized for vibration measurement. Sensitivity, resolution, measuring range, and linearity are among the main criteria. Researchers are trying to improve the performance of a TENG as a self-powered vibration sensor, according to those criteria. Among challenges that are faced when utilizing a TENG as a vibration sensor are the nonlinearity and low resolution. To overcome the nonlinearity problem, the system should be designed with a lateral dimension much larger than their vertical-separation distance. For example, the electricity generation has a linear relationship with the moving distance of the freestanding layer when working in contact-separation mode. Moreover, using grated structures can introduce high-resolution vibration sensing.

Although, high output power is the main criterion for a TENG as a vibration scavenger, some TENG vibration sensors can produce high output power. They can work as effective self-powered sensors as well as energy harvesters. Some of them are reviewed in this work.

## Figures and Tables

**Table 1:** Summary of the reviewed TENGs for vibration energy harvesting showing their main features, dimensions, and output performance.

TENG Energy Harvester	Only TENG/Hybrid	TENG Mode	TENG Materials	Dimensions	Input Excitation	Electrical Output
3D Stack Integrated (Yang et al.) [64]	TENG	Contact- separation	PTFE/Al	231.67 cm^3^	40 Hz	$I_{\mathrm{sc}}=1.14\;\mathrm{mA},\;V_{\mathrm{oc}}=303\;V,\;P_{d}=104.6\;W\;m^{-2}$ @ 2 MΩ
QZS-TENG (Wang et al.) [65]	TENG	Sliding	Bonded glass/ metal	≅471.24 cm^3^	3 Hz	P ≅ 4.06 mW
3D-TENG (Yang et al.) [66]	TENG	Contact- separation + in plane-sliding	PTFE/Al	≅23.56 cm^3^	6 m/s^2^ and 36 Hz	V = 123 V, I = 21 µA
CIT-TENG (Bhatia et al.) [67]	TENG	Contact- separation (impact)	PTFE/Al	25 × 7 × 5.5 cm^3^	0.2g m/s^2^@ 40 Hz range	5 V and 0.75 μA from air compressor
Gas-enhanced (Lv et al.) [68]	TENG	Contact- separation	FEP/ conductive fabric	5 × 5 × 5 cm^3^	3 Hz	V_o_ = 500 V, I_sc_ = 11 µA, Max. P = 0.8 W @ 8 MΩ
Marine pipe EH (Li et al.) [69]	TENG	Contact- separation	PTFE and nylon/ nylon	5 × 5 × 1 cm^3^	8 Hz and 4 mm	V=140 V, Max. P=14 µW @ 200 MΩ
PNG-EMG-TENG (Hemojit et al.) [70]	Hybrid	Contact- separation	PTFE/gold	6 × 6 × 6 cm^3^	ND	V_oc_ = 192 V, I_sc_ = 2.78 mA
PET (He et al.) [71]	Hybrid	Contact- separation	Patterned PDMS/steel	Ø 51 × H 37 mm^2^	0.5 g m/s^2^ and 20 Hz	P= 41 µW, V = 12.6 V @ 800.1 KΩ
TPE (He et al.) [72]	Hybrid	Contact- separation	SR + CNT	Ø 48 × H 27 mm^2^	20 Hz	V = 2V, I = 1 mA.
MFKI-HNG (Toyabur et al.) [40]	Hybrid	Contact- separation	PTFE/Al	Ø 26 × H 50.5 mm^2^	1g m/s^2^ and 5 Hz	$P_{d}\;$= 3.67 mW/cm^3^
EMG-TENG (Zhu et al.) [73]	Hybrid	Contact- separation	PTFE/CB film	6 × 2 × 1 cm^3^	1g m/s^2^ and 18 Hz	TENG: 30 µW and EMG: 53 µW
Polymer-based EMG-TENG (Kumar et al.) [74]	Hybrid	Contact- separation	PTFE/ITO	2 × 2 × 1 cm^3^	0.1–2 g m/s^2^	TENG: 4.8 µW/cm^3^, EMG: 6 µW/cm^3^ (max)
DETEG (Haroun et al.) [75]	Hybrid	Contact- separation	PTFE/Al	8 × 7 × 6 cm^3^	5 Hz	$P_{d}$= 500 W/m^2^

**Table 2:** Summary of the reviewed TENG vibration sensors, which shows their main features and performance.

Sensor	Sensor Type	TENG Mode	Triboelectric Materials	Dimensions	Input Excitation	Performance
CF-TENG (Wang et al.) [80]	Displacement	Contact- separation	FEP/Al	200 cm^3^	15 Hz and 3.5 μm	I_sc_ = 10 nA, V_oc_ = 0.54 V
Micro-grating motion sensor (Zhou et al.) [81]	Displacement	Sliding	$\mathrm{Parylene}/\mathrm{Si}O_{2}$	≅0.18 cm^3^	1 Hz	Resolution = 173 nm
FTENG (Yu et al.) [82]	Displacement	Sliding	FEP/Cu	Ø25 mm × ≅ 65 mm	3 Hz	Sensitivity = 0.391 Vs^2^ m^−1^
AJP motion sensor (Jing et al.) [83]	Displacement	Sliding	PVDF-TrFE/Ag	(25 × 7 × 5.5) cm^3^	ND	Max. sensitivity = 630 μV/μm
AC/DC-TENG (Li et al.) [84]	Displacement	Sliding	FEP/NA	Electrodes: (20 × 20) mm^2^	2 Hz and 5 mm	V_o_ = 3 V
Liquid-metal-based TENG (Zhang et al.) [87]	Acceleration	Contact- separation	PVDF/Hg	(30 × 30 × 6) mm^3^	60 m/s^2^	I_sc_ = 300 nA, V_oc_ = 15.5 V, sensitivity = 0.26 V·s/m^2^
MFMS (Wu et al.) [88]	Acceleration	Sliding	PTFE/NdFeB	(Ø 40 × H 19) mm^2^. V = 23.88 cm^3^	ND	ND
High-g sensor (Dai et al.) [89]	Acceleration	Contact- separation	PDMS/Cu	(14 × 14 × 8) mm^3^	ND	Sensitivity = 1.8 mV/g @ 200 μm beam thickness
MMS (Wang et al.) [90]	Acceleration	Contact- separation	PU/PTFE	ND	50 Hz and 3 m s^−2^	V_o_ = 330 mV
Elastic-beam TENG (Chen et al.) [91]	Acceleration	Contact- separation	PTFE/stainless steel foil (SSF)	(7.5 × 60) mm^2^		Sensitivity = 900 V N^−1^
Inertial sensor (Adly et al.) [92]	Acceleration	Contact- separation	Teflon/Al	(200 × 50 × 20) mm^3^	30 Hz	V_o_ = 6.2 V
V-Q-a TENG (Liu et al.) [93]	Acceleration	Contact- separation	ITO/PET	(3.5 × 6) cm^2^	1–11 m/s^2^	$P_{d}$ = 371.8 mW/m^2^ sensitivity = 20.4 V/(m/s^2^)

## Data Availability

No new data were created or analyzed in this study. Data sharing is not applicable to this article.

## References

[1] Sojan S., Kulkarni R.K. (2016). A Comprehensive Review of Energy Harvesting Techniques and Its Potential Applications. Int. J. Comput. Appl..

[2] Beeby S.P., Tudor M.J., White N.M. (2006). Energy harvesting vibration sources for microsystems applications. Meas. Sci. Technol..

[3] Fang L.H., Hassan S.I.S., Rahim R.B.A., Malek M.F.A. (2016). A study of vibration energy harvester. ARPN J. Eng. Appl. Sci..

[4] Kim W., Bhatia D., Hwang H.J., Choi K., Choi D. (2019). Double impact triboelectric nanogenerators for harvesting broadband vibrations from vehicles. Funct. Compos. Struct..

[5] Zhu Q., Guan M., He Y. Vibration energy harvesting in automobiles to power wireless sensors. Proceedings of the IEEE International Conference on Information and Automation, ICIA.

[6] Carder D.S. (1937). Observed vibrations of bridges. Bull. Seismol. Soc. Am..

[7] Xabibulla G., Gazaloy J., Nishonova G. (2021). Causes and extinguishing equipment of vibrations occurred by machinery and mechanisms. Sci. Prog..

[8] Homayounfar S.Z., Andrew T.L. (2019). Wearable Sensors for Monitoring Human Motion: A Review on Mechanisms, Materials, and Challenges. SLAS Technol..

[9] Choi Y.M., Lee M.G., Jeon Y. (2017). Wearable biomechanical energy harvesting technologies. Energies.

[10] Safaei M., Sodano H.A., Anton S.R. (2019). A review of energy harvesting using piezoelectric materials: State-of-the-art a decade later (2008–2018). Smart Mater. Struct..

[11] Xu J., Tang J. (2015). Multi-directional energy harvesting by piezoelectric cantilever-pendulum with internal resonance. Appl. Phys. Lett..

[12] Wang L., Tong X., Yang H., Wei Y., Miao Y. (2019). Design and analysis of a hollow triangular piezoelectric cantilever beam harvester for vibration energy collection. Int. J. Pavement Res. Technol..

[13] Chilabi H.J., Salleh H., Supeni E.E., As’arry A.B., Rezali K.A.M., Atrah A.B. (2020). Harvesting energy from planetary gear using piezoelectric material. Energies.

[14] Wang C., Zhang Q., Wang W., Feng J. (2018). A low-frequency, wideband quad-stable energy harvester using combined nonlinearity and frequency up-conversion by cantilever-surface contact. Mech. Syst. Signal Process..

[15] Song H.C., Kumar P., Maurya D., Kang M.G., Reynolds W.T., Jeong D.Y. (2017). Ultra-low resonant piezoelectric MEMS energy harvester with high power density. J. Microelectromech. Syst..

[16] Wu J., Hu Z., Gao X., Cheng M., Zhao X., Su W., Li X., Wang Z., Zhou Z., Dong S. (2019). Electrode shape dependence of the barbell-shaped magneto-mechano-electric energy harvester for low-frequency applications. Sens. Actuators A Phys..

[17] Wang C., Zhang Q., Wang W. (2017). Low-frequency wideband vibration energy harvesting by using frequency up-conversion and quin-stable nonlinearity. J. Sound Vib..

[18] Fan K., Cai M., Liu H., Zhang Y. (2019). Capturing energy from ultra-low frequency vibrations and human motion through a monostable electromagnetic energy harvester. Energy.

[19] Fan K., Zhang Y., Liu H., Cai M., Tan Q. (2019). A nonlinear two-degree-of-freedom electromagnetic energy harvester for ultra-low frequency vibrations and human body motions. Renew. Energy.

[20] Haroun A., Yamada I., Warisawa S. (2015). Micro electromagnetic vibration energy harvester based on free/impact motion for low frequency-large amplitude operation. Sens. Actuators A Phys..

[21] Haroun A., Yamada I., Warisawa S. (2015). Study of electromagnetic vibration energy harvesting with free/impact motion for low frequency operation. J. Sound Vib..

[22] Chae S.H., Ju S., Choi Y., Chi Y.E., Ji C.H. (2017). Electromagnetic linear vibration energy harvester using sliding permanent magnet array and ferrofluid as a lubricant. Micromachines.

[23] Ambrozkiewicz B., Litak G., Wolszczak P. (2020). Modelling of electromagnetic energy harvester with rotational pendulum using mechanical vibrations to scavenge electrical energy. Appl. Sci..

[24] Kecik K., Mitura A. (2020). Energy recovery from a pendulum tuned mass damper with two independent harvesting sources. Int. J. Mech. Sci..

[25] Basset P., Galayko D., Paracha A.M., Marty F., Dudka A., Bourouina T. (2009). A batch-fabricated and electret-free silicon electrostatic vibration energy harvester. J. Micromech. Microeng..

[26] Sidek O., Khalid M.A., Ishak M.Z., Miskam M.A. Design and simulation of SOI-MEMS electrostatic vibration energy harvester for micro power generation. Proceedings of the ECCE—International Conference on Electrical, Control and Computer Engineering.

[27] Dudka A., Basset P., Cottone F., Blokhina E., Galayko D. (2013). Wideband electrostatic vibration energy harvester (e-VEH) having a low start-up voltage employing a high-voltage integrated interface. J. Phys..

[28] Dragunov V., Dorzhiev V. (2013). Electrostatic vibration energy harvester with increased charging current. J. Phys..

[29] Dorzhiev V., Karami A., Basset P., Dragunov V., Galayko D. (2014). MEMS electrostatic vibration energy harvester without switches and inductive elements. J. Phys..

[30] Basset P., Galayko D., Cottone F., Guillemet R., Blokhina E., Marty F., Bourouina T. (2014). Electrostatic vibration energy harvester with combined effect of electrical nonlinearities and mechanical impact. J. Micromech. Microeng..

[31] Wu C., Wang A.C., Ding W., Guo H., Wang Z.L. (2019). Triboelectric Nanogenerator: A Foundation of the Energy for the New Era. Adv. Energy Mater..

[32] Bera B. (2016). Literature Review on Triboelectric Nanogenerator. Imp. J. Interdiscip. Res..

[33] Zhang H., Yao L., Quan L., Zheng X. (2020). Theories for triboelectric nanogenerators: A comprehensive review. Nanotechnol. Rev..

[34] Niu S., Wang S., Lin L., Liu Y., Zhou Y.S., Hu Y., Wang Z.L. (2013). Theoretical study of contact-mode triboelectric nanogenerators as an effective power source. Energy Environ. Sci..

[35] Lin Z., Chen J., Yang J. (2016). Recent Progress in Triboelectric Nanogenerators as a Renewable and Sustainable Power Source. J. Nanomater..

[36] Wu C., Huang H., Li R., Fan C. (2020). Research on the potential of spherical triboelectric nanogenerator for collecting vibration energy and measuring vibration. Sensors.

[37] Zhang H., Yang Y., Su Y., Chen J., Adams K., Lee S., Hu C., Wang Z.L. (2014). Triboelectric nanogenerator for harvesting vibration energy in full space and as self-powered acceleration sensor. Adv. Funct. Mater..

[38] Xu M., Wang P., Wang Y.C., Zhang S.L., Wang A.C., Zhang C., Wang Z., Pan X., Wang Z.L. (2018). A Soft and Robust Spring Based Triboelectric Nanogenerator for Harvesting Arbitrary Directional Vibration Energy and Self-Powered Vibration Sensing. Adv. Energy Mater..

[39] Zhang Z., Cai J. (2020). High output triboelectric nanogenerator based on PTFE and cotton for energy harvester and human motion sensor. Curr. Appl. Phys..

[40] Rahman M.T., Sohel Rana S., Salauddin M., Maharjan P., Bhatta T., Kim H., Park J.Y. (2020). A highly miniaturized freestanding kinetic-impact-based non-resonant hybridized electromagnetic-triboelectric nanogenerator for human induced vibrations harvesting. Appl. Energy.

[41] Wang X., Wen Z., Guo H., Wu C., He X., Lin L., Wang Z.L. (2016). Fully Packaged Blue Energy Harvester by Hybridizing a Rolling Triboelectric Nanogenerator and an Electromagnetic Generator. ACS Nano.

[42] Hu Y., Yang J., Jing Q., Niu S., Wu W., Wang Z.L. (2013). Triboelectric nanogenerator built on suspended 3D spiral structure as vibration and positioning sensor and wave energy harvester. ACS Nano.

[43] Zeng Q., Wu Y., Tang Q., Liu W., Wu J., Zhang Y., Yin G., Yang H., Yuan S., Tan D. (2020). A high-efficient breeze energy harvester utilizing a full-packaged triboelectric nanogenerator based on flow-induced vibration. Nano Energy.

[44] Lu S., Gao L., Chen X., Tong D., Lei W., Yuan P., Mu X., Yu H. (2020). Simultaneous energy harvesting and signal sensing from a single triboelectric nanogenerator for intelligent self-powered wireless sensing systems. Nano Energy.

[45] He W., Fu X., Zhang D., Zhang Q., Zhuo K., Yuan Z., Ma R. (2021). Recent progress of flexible/wearable self-charging power units based on triboelectric nanogenerators. Nano Energy.

[46] Li G.Z., Wang G.G., Cai Y.W., Sun N., Li F., Zhou H.L., Zhao H.X., Zhang X.N., Han J.C., Yang Y. (2020). A high-performance transparent and flexible triboelectric nanogenerator based on hydrophobic composite films. Nano Energy.

[47] Bertacchini A., Larcher L., Lasagni M., Pavan P. Ultra-low cost triboelectric energy harvesting solutions for embedded sensor systems. Proceedings of the IEEE-NANO—15th International Conference on Nanotechnology.

[48] Jang S., Kim H., Oh J.H. (2017). Simple and rapid fabrication of pencil-on-paper triboelectric nanogenerators with enhanced electrical performance. Nanoscale.

[49] Jing T., Xu B., Yang Y., Li M., Ga Y. (2020). Organogel electrode enables highly transparent and stretchable triboelectric nanogenerators of high power density for robust and reliable energy harvesting. Nano Energy.

[50] Zhu J., Zhu M., Shi Q., Wen F., Liu L., Dong B., Haroun A., Yang Y., Vachon P., Guo X. (2020). Progress in TENG technology—A journey from energy harvesting to nanoenergy and nanosystem. EcoMat.

[51] Haroun A., Le X., Gao S., Dong B., He T., Zhang Z., Wen F., Xu S., Lee C. (2021). Progress in micro/nano sensors and nanoenergy for future AIoT-based smart home applications. Nano Express.

[52] Xie L., Zhai N., Liu Y., Wen Z., Sun X. (2021). Hybrid Triboelectric Nanogenerators: From Energy Complementation to Integration. Research.

[53] Vidal J.V., Slabov V., Kholkin A.L., dos Santos S.M.P. (2021). Hybrid Triboelectric-Electromagnetic Nanogenerators for Mechanical Energy Harvesting: A Review. Nano-Micro Lett..

[54] Ha M., Canon Bermudez G.S., Kosub T., Mönch I., Zabila Y., Oliveros Mata E.S., Illing R., Wang Y., Fassbender J., Makarov D. (2021). Printable and Stretchable Giant Magnetoresistive Sensors for Highly Compliant and Skin-Conformal Electronics. Adv. Mater..

[55] Dionisio R., Torres P., Ramalho A., Ferreira R. (2021). Magnetoresistive sensors and piezoresistive accelerometers for vibration measurements: A comparative study. J. Sens. Actuator Netw..

[56] Qian S., Qin L., He J., Zhang N., Qian J., Mu J., Geng W., Hou X., Chou X. (2020). A lead-free stretchable piezoelectric composite for human motion monitoring. Mater. Lett..

[57] Gao Q., Cheng T., Wang Z.L. (2021). Triboelectric mechanical sensors—Progress and prospects. Extreme Mech. Lett..

[58] Du T., Zuo X., Dong F., Li S., Mtui A.E., Zou Y., Zhang P., Zhao J., Zhang Y., Sun P. (2021). A self-powered and highly accurate vibration sensor based on bouncing-ball triboelectric nanogenerator for intelligent ship machinery monitoring. Micromachines.

[59] Chen J., Wang Z.L. (2017). Reviving Vibration Energy Harvesting and Self-Powered Sensing by a Triboelectric Nanogenerator. Joule.

[60] Lin Z., Long W., Chen L.J., Niu S., Zi Y. (2016). Green Energy and Technology Triboelectric Nanogenerators.

[61] Niu S., Liu Y., Chen X., Wang S., Zhou Y.S., Lin L., Xie Y., Wang Z.L. (2015). Theory of freestanding triboelectric-layer-based nanogenerators. Nano Energy.

[62] Liu C., Zhang N., Li J., Dong L., Wang T., Wang Z., Wang G., Zhou X., Zhang J. (2019). Harvesting ultralow frequency (<1 Hz) mechanical energy using triboelectric nanogenerator. Nano Energy.

[63] He J., Fan X., Mu J., Wang C., Qian J., Li X., Hou X., Geng W., Wang X., Chou X. (2020). 3D full-space triboelectric-electromagnetic hybrid nanogenerator for high-efficient mechanical energy harvesting in vibration system. Energy.

[64] Yang W., Chen J., Jing Q., Yang J., Wen X., Su Y., Zhu G., Bai P., Wang Z.L. (2014). 3D stack integrated triboelectric nanogenerator for harvesting vibration energy. Adv. Funct. Mater..

[65] Wang K., Zhou J., Ouyang H., Chang Y., Xu D. (2021). A dual quasi-zero-stiffness sliding-mode triboelectric nanogenerator for harvesting ultralow-low frequency vibration energy. Mech. Syst. Signal Process..

[66] Yang J., Chen J., Yang Y., Zhang H., Yang W., Bai P., Su Y., Wang Z.L. (2013). Broadband vibrational energy harvesting based on a triboelectric nanogenerator. Adv. Energy Mater..

[67] Bhatia D., Hwang H.J., Huynh N.D., Lee S., Lee C., Nam Y., Kim J.G., Choi D. (2019). Continuous scavenging of broadband vibrations via omnipotent tandem triboelectric nanogenerators with cascade impact structure. Sci. Rep..

[68] Lv S., Yu B., Huang T., Yu H., Wang H., Zhang Q., Zhu M. (2018). Gas-enhanced triboelectric nanogenerator based on fully-enclosed structure for energy harvesting and sensing. Nano Energy.

[69] Li R., Zhang H., Wang L., Liu G. (2021). A Contact-Mode Triboelectric Nanogenerator for Energy. Sensors.

[70] Singh H.H., Kumar D., Khare N. (2020). A synchronous piezoelectric-triboelectric-electromagnetic hybrid generator for harvesting vibration energy Sustain. Energy Fuels.

[71] He X., Wen Q., Sun Y., Wen Z. (2017). A low-frequency piezoelectric-electromagnetic-triboelectric hybrid broadband vibration energy harvester. Nano Energy.

[72] He J., Wen T., Qian S., Zhang Z., Tian Z., Zhu J., Mu J., Hou X., Geng W., Cho J. (2018). Triboelectric-piezoelectric-electromagnetic hybrid nanogenerator for high-efficient vibration energy harvesting and self-powered wireless monitoring system. Nano Energy.

[73] Zhu J., Wang A., Hu H., Zhu H. (2017). Hybrid electromagnetic and triboelectric nanogenerators with multi-impact for wideband frequency energy harvesting. Energies.

[74] Gupta R.K., Shi Q., Dhakar L., Wang T., Heng C.H., Lee C. (2017). Broadband energy harvester using non-linear polymer spring and electromagnetic/triboelectric hybrid mechanism. Sci. Rep..

[75] Haroun A., Lee C. (2022). Dielectric-elastomer-enhanced triboelectric nanogenerator with amplified outputs. Sens. Actuators A Phys..

[76] Hassani F.A., Shi Q., Wen F., He T., Haroun A., Yang Y., Feng Y., Lee C. (2020). Smart materials for smart healthcare—Moving from sensors and actuators to self-sustained nanoenergy nanosystems. Smart Mater. Med..

[77] Haroun A., Yamada I., Warisawa S. (2016). Investigation of Kinetic Energy Harvesting from Human Body Motion Activities Using Free/Impact Based Micro Electromagnetic Generator. Diabetes Cholest. Metab..

[78] Seol M.L., Han J.W., Park S.J., Jeon S.B., Choi Y.K. (2016). Hybrid energy harvester with simultaneous triboelectric and electromagnetic generation from an embedded floating oscillator in a single package. Nano Energy.

[79] Salauddin M., Toyabur R.M., Maharjan P., Park J.Y. (2018). High performance human-induced vibration driven hybrid energy harvester for powering portable electronics. Nano Energy.

[80] Wang S., Niu S., Yang J., Lin L., Wang Z.L. (2014). Quantitative measurements of vibration amplitude using a contact-mode freestanding triboelectric nanogenerator. ACS Nano.

[81] Zhou Y.S., Zhu G., Niu S., Liu Y., Bai P., Jing Q., Wang Z.L. (2013). Nanometer resolution self-powered static and dynamic motion sensor based on micro-grated triboelectrification. Adv. Mater..

[82] Yu H., He X., Ding W., Hu Y., Yang D., Lu S., Wu C., Zou H., Liu R., Lu C. (2017). A Self-Powered Dynamic Displacement Monitoring System Based on Triboelectric Accelerometer. Adv. Energy Mater..

[83] Jing Q., Choi Y.S., Smith M., Ćatić N., Ou C., Kar-Narayan S. (2019). Aerosol-Jet Printed Fine-Featured Triboelectric Sensors for Motion Sensing. Adv. Mater. Technol..

[84] Li S., Liu D., Zhao Z., Zhou L., Yin X., Li X., Gao Y., Zhang C., Zhang Q., Wang J. (2020). A Fully Self-Powered Vibration Monitoring System Driven by Dual-Mode Triboelectric Nanogenerators. ACS Nano.

[85] Liu D., Yin X., Guo H., Zhou L., Li X., Zhang C., Wang J., Wang Z.L. (2019). A constant current triboelectric nanogenerator arising from electrostatic breakdown. Sci. Adv..

[86] Liu C., Fang L., Zou H., Wang Y., Chi J., Che L., Zhou X., Wang Z., Wang T., Dong L. (2021). Theoretical investigation and experimental verification of the self-powered acceleration sensor based on triboelectric nanogenerators (TENGs). Extreme Mech. Lett..

[87] Zhang B., Zhang L., Deng W., Jin L., Chun F., Pan H., Wang Z.L. (2017). Self-Powered Acceleration Sensor Based on Liquid Metal Triboelectric Nanogenerator for Vibration Monitoring. ACS Nano.

[88] Wu Z., Ding W., Dai Y., Dong K., Wu C., Zhang L., Lin Z., Cheng J., Wang Z.L. (2018). Self-Powered Multifunctional Motion Sensor Enabled by Magnetic-Regulated Triboelectric Nanogenerator. ACS Nano.

[89] Dai K., Wang X., Yi F., Jiang C., Li R., You Z. (2018). Triboelectric nanogenerators as self-powered acceleration sensor under high-g impact. Nano Energy.

[90] Chen Y., Wang Y.-C., Zhang Y., Zou H., Lin Z., Zhang G., Zou C., Wang Z.L. (2018). Elastic-Beam Triboelectric Nanogenerator for High-Performance Multifunctional Applications: Sensitive Scale, Acceleration/Force/Vibration Sensor, and Intelligent Keyboard. Adv. Energy Mater..

[91] Wang Z., Zhang F., Li N., Yao T., Lv D., Cao G. (2020). Self-Powered Multifunctional Triboelectric Sensor Based on PTFE/PU for Linear, Rotary, and Vibration Motion Sensing. Adv. Mater. Technol..

[92] Adly M.A., Arafa M.H., Hegazi H.A. (2021). Modeling and optimization of an inertial triboelectric motion sensor. Nano Energy.

[93] Liu C., Wang Y., Zhang N., Yang X., Wang Z., Zhao L., Yang W., Dong L., Che L., Wang G. (2020). A self-powered and high sensitivity acceleration sensor with V-Q-a model based on triboelectric nanogenerators (TENGs). Nano Energy.

[94] Xiao X., Zhang X., Wang S., Ouyang H., Chen P., Song L., Yuan H., Ji Y., Wang P., Li Z. (2019). Honeycomb Structure Inspired Triboelectric Nanogenerator for Highly Effective Vibration Energy Harvesting and Self-Powered Engine Condition Monitoring. Adv. Energy Mater..

[95] Wang L., He T., Zhang Z., Zhao L., Lee C., Luo G., Mao Q., Yang P., Lin Q., Li X. (2021). Self-sustained autonomous wireless sensing based on a hybridized TENG and PEG vibration mechanism. Nano Energy.

[96] Kim G.W., Kim J. (2013). Compliant bistable mechanism for low frequency vibration energy harvester inspired by auditory hair bundle structures. Smart Mater. Struct..

[97] Li Z., Bai S., Madsen O., Chen W., Zhang J. (2020). Design, modeling and testing of a compact variable stiffness mechanism for exoskeletons. Mech. Mach. Theory.

